# Epidemiology of Inflammatory Bowel Diseases in Iran and Asia; A Mini Review

**Published:** 2013-06

**Authors:** Ali Reza Safarpour, Seyed Vahid Hosseini, Davood Mehrabani

**Affiliations:** 1 Colorectal Research Center, Faghihi Hospital, Shiraz University of Medical Sciences, Shiraz, Iran;; 2 Laparoscopy Research Center, Faghihi Hospital, Shiraz University of Medical Sciences, Shiraz, Iran;; 3 Stem Cell and Transgenic Technology Research Center, Department of Pathology, Shiraz University of Medical Sciences, Shiraz, Iran

**Keywords:** Inflammatory bowel disease, Epidemiology, Prevalence, Iran

## Abstract

The prevalence of inflammatory bowel diseases (IBDs) is set to stabilize in Western Europe and North America, as opposed to its increasing trend in developing countries in Asia. The epidemiology of IBDs in areas where the incidence and prevalence are relatively low provides an opportunity for researchers to determine the unknown aspects of them. In this review article, the PubMed and MEDLINE databases were searched from 1970 to 2012 and the epidemiological aspects assessed in Iranian articles were compared with identical subjects in other Asian countries. During this period, there were 21 documented articles on IBD epidemiology in Iran and 52 in Asia. According to the present review, CTLA-gene polymorphism and male/female ratio in ulcerative colitis (UC), incidence of extra-intestinal manifestations, extent of intestinal involvement, and family history in both UC and Crohn’s disease (CD) seemed to be different between Asia and Iran. In contrast, the incidence of primary sclerosing cholangitis in IBD patients and association between NO2/CARD15 mutation and CD as C3435-T allele and UC were nearly the same. The rate of IBD has increased significantly in Iran, as has that of other Asian countries during the last decade. A thorough, well-designed, population-based, multi-regional epidemiologic study seems mandatory due to the substantial demographic and characteristic variability in IBD patients in our region.

## Introduction

Inflammatory bowel diseases (IBDs) are a group (ulcerative colitis [UC] and Crohn’s disease [CD]) of digestive system diseases whose causes are not completely clarified.^1^^,^^2^ Environment, genetics, and immune factors affect the occurrence of IBDs; and since 1950, the incidence has rapidly increased in Northern Europe and North America.^3^ It seems that while the prevalence of IBDs is set to stabilize in Western Europe and North America, it has an increasing trend in South America, Asia, and Pacific regions.^4^ Meanwhile, geographical, racial, genetic, sexual, and habitual differences have provided a basis for epidemiological studies.^5^ The recent rising trend in these diseases in Asia is probably similar to that in western countries in the past decades.^4^ The epidemiological research of IBDs in the areas in which the incidence and prevalence are relatively low (compared with northern countries) provides an opportunity for researchers to determine the hitherto unknown aspects of the disease such as pathogenesis, etiology, and risk factors; all of which can be beneficial for decision-makers in economic and health sectors.^5^^-^^7^

There are some limitations in epidemiological studies in Asian countries-including lack of an organized registry and follow-up center, absence of an appropriate design in population-based studies in an expanded level, nonexistence of a standard system in the definition and registry of diseases, and dearth of valid information and design in most hospital-based studies versus population-based ones.^8^

The studies conducted thus far in developed countries have shown that prospective and population-based studies have a higher incidence rate of IBDs than retrospective and hospital-based studies.^9^ Iran, as one of the largest Asian countries in the Middle East with cultural and ethnic variation on the one hand and adjacency to Asian parts of Turkey, Persian Gulf region, Central Asia, Pakistan, and Afghanistan on the other hand, is fertile ground for investigation into the epidemiology of rare diseases in general and IBDs in particular.^10^

The present review aimed to study the epidemiology of IBDs in Iran in comparison to Asian countries. There have been several epidemiologic studies on IBDs in Iran with respect to such variables as age, gender, family history, common risk factors (e.g. genetics, family aggregation, appendectomy, and smoking), less common risk factors, and clinical features. In each section of this review, data on IBDs Iran will be compared with those in Asian countries.

## Materials and Methods

PubMed, Medline, and Persian databases-including SID and IranMedex were searched from 1970 to 2012. The keywords used in this search were inflammatory bowel disease, Iran, ulcerative colitis, Crohn’s disease, epidemiology, risk factors, genetics, extra-intestinal manifestations, Asia, Middle East, and ethnicity. OR, AND or NOT were applied during search by MeSH, appropriately. Due to restrictions, only the Persian and English languages were used as limitation (Persian for references in Iran). 

**Figure 1:**
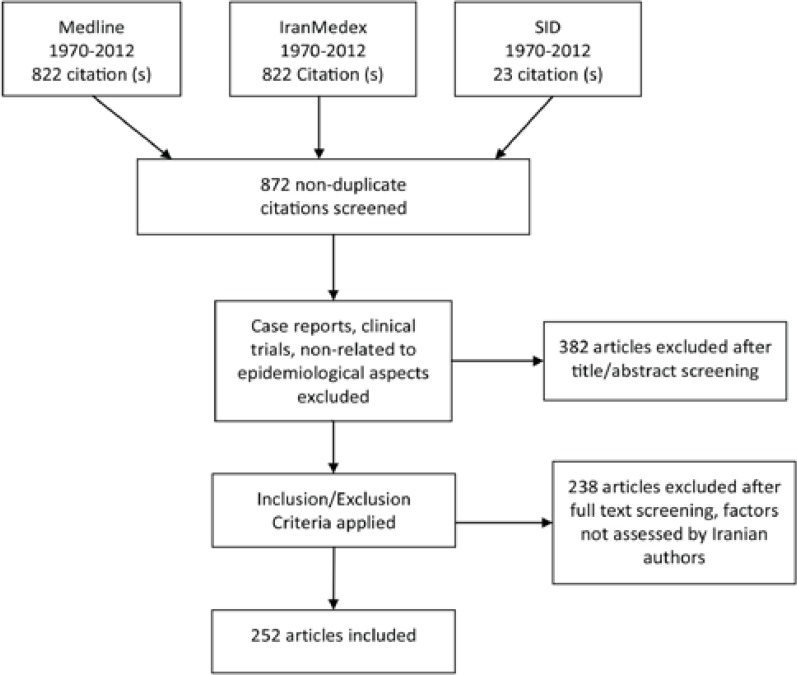
This flow chart depicts the inclusion and exclusion criteria applied in the present review

Only the epidemiological aspects assessed in Iranian articles were compared with the same subjects in other Asian countries. Articles in the form of clinical trials, case reports, case series, and radiologic and surgical procedures were excluded. Each article was surveyed twice by two authors, and the obtained data were recorded in a pre-prepared checklist. Of all the articles on the subject in Iran (available in above indices), two were duplicated and just one was used in the present study. Asian countries were defined according to the latest confirmed map by the United Nations (UN) (United Nations Statistics Division, 2011). Among the articles, only those review articles whose references were used in our references were selected in order to complete our reference list.^10^ In total, there were 21 documented articles on IBD epidemiology in Iran and 170 in Asia. The articles will be described in the following section (figure 1).

*Incidence and Prevalence*

According to a recent systematic review that has assessed the trend of incidence and prevalence of IBDs around the world, the incidence and prevalence rates of IBDs have increased in the last 4-5 decades. The annual incidence rates were 0.6-24.3, 0.1-6.3, and 0-19.2 per 100,000 individuals for UC and 0.3-12.7, 0.04-5.0, and 0-20.2 per 100,000 individuals for CD in Europe, Asia and Middle East, and North America-respectively. Also, the prevalence ranges were 4.9-505, 4.9-168.3, and 37.5-248.6 per 100,000 persons for UC and 0.6-322, 0.88-67.9, and 16.7-318.5 per 100,000 persons for CD in Europe, Asia and Middle East, and North America-respectively.^11^

We were not able to conduct a precise study on the incidence and prevalence of IBDs in Iran due to the absence of national registries and population-based studies.^12^ Iran does not have as high a prevalence rate of IBDs as do western countries; however, due to changes in people’s lifestyle and industrialization in tandem with other Asian countries, we may expect a rising trend in our region.^13^ Indeed, a proliferation in the number of published articles on IBDs during the last decade is evidence of the vigorous elevation of concerns over IBDs in Iran: where there were only 3 articles on IBDs before the year 2000 in Iran, the period between 2000 and 2012 saw the figure soar to 26 articles.

A study in South Korea showed that the prevalence of UC was 7.57 in 100,000 individuals in 1997, whereas an increase of 30.87 patients in 100,000 individuals was noted in 2005.^14^ This rising trend is also visible in Japan. The prevalence of CD, which was 2.9 in 100,000 people in Japan in 1986, reached 13.5 in 1998.^15^ The prevalence of IBDs in the Middle East countries such as Lebanon^16^ and Israel^17^ also indicates a growing trend. The prevalence of UC in Kuwait in 1999 was 41.7 for 100,000 individuals.^18^ The annual incidence rates of UC and CD were 3.08 and 1.34 cases per 100,000 person-years in South Korea,^14^ 1.95 and 0.51 in Japan,^19^ 4.1 and 1.4 in Lebanon,^17^ and 5.04 and 5.0 cases per 100,000 person-years in Kibbutz, Israel^17^ respectively. A population-based study in Punjab, North India, demonstrated that the prevalence of UC was 44.3 in 100,000 persons and the incidence of this disease was 6.02 per 100,000 person-years.^20^

*Demographic Variables: Gender*

Gender assessment on IBDs in Iran illustrates male/female (M/F) ratios for UC of 1.6/1,^21^ 0.78/1.0,^12^ 0.7/1.0,^22^ 0.8/1.0,^23^ and 1.2/1.1,^24^ and M/F ratios for CD of 1.4/1.0,^21^ 1.18/1.0,^12^ 0.9/1.0,^22^ 1.2/.8,^24^ and 1.3/1.0.^23^ It seems that female predominance in UC and male predominance in CD are the major demographic patterns of IBDs in Iran. The male predominance has been reported for CD in China,^25^ Japan,^15^ and Korea.^14^ The M/F ratio for UC is nearly equal in Korea and Japan^26^ and the F/M ratio is 1.33 in Riyadh, Saudi Arabia.^27^

*Age*

The mean age at diagnosis of IBDs in Iranian patients is identical to that of other Asian countries; while in four different studies, it was 33.6 for UC^12^^,^^23^^,^^28^ and 32.3 for CD.^12^^,^^23^ One peak age of onset has been reported in the second decade of life and the second peak has not been seen in Iran.^12^^,^^22^^,^^23^^,^^29^ Based on one report, Asian countries have a peak age of onset at 20-39 years of age for both diseases and the second peak has not been seen in most of them; whereas a small second peak has been reported by the same author in another study.^14^ The trend of the second peak has also been observed in a study conducted in the Chinese population of Hong Kong.^25^

*Urban Versus Rural Distribution*

This factor has been assessed in three studies in Iran. The mean percentage of UC in urban areas was reported to be 73.8%, whereas this mean percentage for CD was 86%^23^^,^^29^ which clearly denotes a higher prevalence rate in city dwellers. In a study in Turkey, the prevalence of UC was low in rural residents in comparison with city dwellers: 2.18 versus 5.87 in 100,000 persons.^30^

*Risk Factors: Genetics*

In a case-control study in Iran,^31^ a significant relationship was seen between C3435-T allele and UC (P=0.001). Also, the frequency of homozygote genotypes (T/T) and heterozygote (C/T) of this allele was significantly higher in a group of patients UC than in a control group (P=0.041 and P=0.044, respectively). In fact, there was a relationship between MDR 1 gene polymorphisms such as C3435T and UC by reducing P-glyco-protein expression.^32^ These results were echoed by a similar study on Chinese and Malaysian patients:^33^ Chinese and Malaysian patients had a higher frequency of C allele than their Indian counterparts (OR: 0.46, 95%CI: 0.39-0.53; OR: 0.48, 95%CI: 0.42-0.55; and OR: 0.38, 95%CI: 0.31-0.45, respectively).

In other case-control studies in Iran,^12^^,^^34^ the relationship between three common types of CARD15/NOD2 gene mutations in IBD patients were evaluated. These three types of mutations were R 702W, G908 R, and 1007fsinsC. The frequency of R 702W was significantly higher in CD patients than in the control group (OR: 19.21, 95%CI: 4.23-87.32; P<0.001). Also, no significant relationship was seen between the frequencies of the other two variants in CD patients and the frequencies of all the three gene mutations in UC patients.

In a similar study in Japan,^35^ no significant correlation was noted between these three common mutations and CD. Conversely, a study conducted in Israel^36^ showed that NOD2/CARD15 mutations in CD patients of Ashkenazi Jews were significantly high. In studies carried out in Turkey^37^ and Hong Kong on Chinese patients,^38^ no significant relationship was observed between the above mutations in CD patients. No significant relationship was seen between the three above mutations and CD in Iranian patients.^39^

The relationship between cytotoxic T lymphocyte-associated Antigen 4 gene polymorphisms (CTLA-4) and UC was evaluated in a case-control study by Lankarani et al. in 2006.^40^ CTLA-4 polymorphism was not associated with UC in the Iranian population. Conversely, a strong relationship was demonstrated between CTLA-4 and UC in China.^41^ The same relationship was seen in Japanese patients.^42^ It seems that there is a difference between the people of East-Asian countries and Iranians in the Middle East as regards the relationship between CTLA4 gene polymorphism and UC. In another case-control study,^43^ a significant difference was observed in the frequency of 2 promotor polymorphisms of the transforming growth factor-ß1 gene, -800G>A and -509c<T between patients with UC and the control group.

*Family History*

Of all the studies reviewed, four revealed positive family history in the relatives of Iranian patients with IBDs. The rates of positive family history in the immediate relatives of UC and CD patients have been reported to be 10.2% and 7.5%, respectively.^22^^,^^23^ Overall, positive family history in first and second-degree relatives was 20.71% in UC patients and 11.92% in CD patients.^14^^,^^18^ Positive family history has been reported more frequently in Iranian UC patients than in their CD counterparts. The above percentage was 1.5 to 5.6% for Chinese patients with UC^44^^,^^45^ and 2.8% for patients with CD in Japan.^26^ Lebanese UC patients had 26.1% and patients with CD had 13.6% rates of positive family history.^16^

*Appendectomy*

Out of three studies on appendectomy as a risk factor in Iranian IBD patients, two descriptive retrospective studies revealed appendectomy rates of 5.5% and 4.6% in UC patients and 17.9% and 15.59% in patients with CD.^12^^,^^22^ In the third case-control study, which was conducted on 382 UC and 46 CD patients as case groups and 382 and 184 individuals as control groups, the significant protective effect of appendectomy on UC was confirmed (OR: 0.38, %95CI: 0.19-0.76; P<0.004). This study also showed a positive correlation between appendectomy and CD as a significant risk factor (OR: 5.49, 95% C1: 1.41-21.34; P=0.02).^46^ The significant protective effect of appendectomy in UC was observed in case-control studies in China and japan.^42^^,^^47^ No significant relationship between CD and appendectomy was seen in a study from Israel.^48^

*Smoking*

An analytical case-control study to evaluate the relationship between smoking and IBDs in Iranian patients showed the significant protective effect of smoking on UC (OR: 0.2, %95 CI: 0.13-0.32; P<0.0001)^49^ and reported no significant relationship between CD and smoking.

The results of descriptive studies in Iran have revealed an absence of smoking in the majority of CD and UC patients.^12^^,^^22^^,^^23^ Water-pipe smoking is another type of smoking which is very common in Iran. This risk factor is not mentioned in related studies. The protective effect of smoking on UC has been observed in two studies in Japan and China.^19^^,^^47^ Some studies, carried out to find the relationship between CD and smoking in Asian countries, have highlighted smoking as a risk factor in patients in Israel.^48^ The nonexistence of a relationship between smoking, as a risk factor, and CD was reported from Hong Kong.^45^

*Less Common Risk Factors*

The risk factors which have been reported less frequently in Asia have not been studied in Iran. These risk factors, whose relationship with IBDs is still controversial-include consumption of oral contraceptive pills (OCPs),^50^ non-steroidal anti-inflammatory drugs (NSAIDs),^51^ antibiotics,^52^ history of breast feeding versus formula feeding,^53^ childhood infections such as measles and mumps,^54^ Clostridium difficile infection,^55^ diet,^56^ pets,^57^ and exposure to fresh vegetables during infancy and childhood.^47^^,^^58^ Research has shown that the consumption of OCPs and NSAIDs has an inverse significant effect on UC for OCPs (OR: 0.32, 95% CI: 0.19-0.53; P<0.001) and for NSAIDs (OR: 0.36, 95% CI: 0.19-0.67; P<0.001). No significant effect has been observed between CD and OCPs or NSAIDs.^6^

In the Malekzadeh and colleagues’ case-control study (2009),^49^ a relationship between early exposure to home refrigeration and CD was reported. Based on the results of this study, exposure to food kept in the refrigerator during infancy was shown as a risk factor for CD (OR: 2.08, 95% CI: 1.01-4.29; P<0.05). Another case-control study conducted in Iran revealed no significant correlation between measles vaccination and breast feeding during infancy and adulthood presentation of UC and CD (P>0.1).^59^ No study was available on other risk factors in Iran.

**Clinical Course and Characteristics of Inflammatory Bowel Diseases: Extent of Diseases**

*Ulcerative Colitis*

The involvement of various parts of the large intestine in Iranian UC patients has been evaluated in six studies. The results of these studies are presented in table 1. It seems that the most frequent pattern of involvement in Iran was proctitis.^60^ This pattern is also dominant in South Korea,^14^ Hong Kong,^61^ and Israel.^17^ The most common pattern was left-sided colitis in China^41^ and Singapore^62^ and pancolitis in Japan,^63^ Lebanon,^16^ and Kuwait.^17^

**Table 1:** Extent of intestinal involvement in Iranian patients with ulcerative colitis

**Authors**	**Year**	**No. of cases**	**Type of study**	Pancolitis* (%)	Proctitis** (%)	Left-sided colitis*** (%)
Mir-Madjlessi et al.^29^	1985	112	Hospital-based	28	42	-
Aghazadeh et al.^23^	2005	401	Hospital-based	18.1	51.9	30.0
Teimoori-Toolabi et al.^39^	2010	89	Hospital-based	48.3	28.0	5.6
Derakhshan et al.^12^	2008	671	Hospital-based	8.85	0.65	90.49
Vahedi et al.^22^	2009	293	Hospital-based	17.0	51.0	32.0

*Pancolitis, all the parts of the colon; **Proctitis, inflammation up to 15 cm from the anterior portion; ***Left-sided colitis, inflammation up to the splenic flexure

*Crohn’s Disease*

The involvement of the sigmoid colon in addition to the mid-part of the ileum was cited in the first report of CD in Iran in 1973. The terminal ileum, however, was free from the disease.^64^ In recent studies, with the exception of one study which reported that the part with the most frequent involvement was the terminal ileum (43.7%),^23^ the involvement of the large intestine, colon, and small intestine was significantly recognized in Iranian patients.^12^^,^^22^ Similar to the pattern in Iran, in Asian countries such as Japan,^65^ China,^66^ and Hong Kong, the small bowel was the least significantly involved portion of the digestive system in CD patients and that the most common pattern was ileocolic involvement.^67^

In a study conducted in Riyadh, Saudi Arabia, 16% of CD patients showed the involvement of small bowel, whereas 78% of them exhibited both small and large bowel involvement.^27^ Lebanese patients with CD were similar to those in Saudi Arabia.^16^

*Extra-Intestinal Manifestations*

A comparison between the frequency of extra-intestinal manifestations (EIMs) in Iran and Asia showed a higher frequency in Iran (tables 2 and 3). The frequency of EIMs was 6.4-44.5% in UC and 16.6-47.4% in CD patients in Iran, while the rate of EIMs was 22% in Chinese^68^ and 23% in South Korean CD patients.^69^ The frequency of EIMs in both diseases (UC and CD) in Kuwait^18^ was 38%; this rate in Chinese and Indian residents in Singapore was 6% and 14% - respectively.^62^

**Table 2:** Extra-intestinal manifestations of ulcerative colitis in Iranian patients (%)

**Authors**	**Sclerosing cholangitis**	**Arthritis**	**Oral Aphtus**	EN*	PG**	**Uveitis**	**Total**
Mir-Madjlessi et al.^29^	2	3.5	-	-	-	0.9	6.4
Aghazadeh et al.^23^	3.9	5.8	5.2	1.0	0.5	-	16.4
Teimoori-Toolabi et al.^39^	6.7	-	-	-	-	-	6.7
Derakhshan et al.^12^	5.5	29.0	-	3.0	1.6	-	39.1
Vahedi et al.^22^	9.5	32.0	2.0	-	1.0	-	44.5

*Erythema nodosum; **Pyoderma gangranosum

**Table 3:** Extra-intestinal manifestations of Crohn’s disease in Iranian patients (%)

**Authors**	**Sclerosing cholangitis**	**Arthritis**	**Oral Aphtus**	EN*	PG**	**Uveitis**	**Total**
Mir-Madjlessi et al.^29^	-	-	-	-	-	-	-
Aghazadeh et al.^23^	0.2	9.0	13.0	2.1	0.4	-	16.6
Teimoori-Toolabi et al.^39^	-	-	-	-	-	-	-
Derakhshan et al.^12^	1.8	35.0	-	6.42	3.66	-	46.88
Vahedi et al.^22^	2.8	37.0	5.3	1.9	0.4	-	47.4

*Erythema nodosum; **Pyoderma gangranosum

*Primary Sclerosing Cholangitis*

In Iranian patients, the overall frequency of primary sclerosing cholangitis was 5.4% in UC and 1.6% in CD patients. In Turkish patients, the rate of this disease was 2.3% in UC and 3.6% in CD patients.^70^

*Pediatric Inflammatory Bowel Diseases*

There were two reports available on pediatric IBDs in Iran: one a hospital-based study and the other a retrospective study.^21^^,^^71^ The former underlined pancolitis as the most common involved site (69.6%) in UC on colonoscopy and reported a higher M/F ratio in both UC (0.6/0.4) and CD (0.58/0.42) patients. In the latter, the most common colonoscopic feature was erythema in UC and ulcer in CD.

In a study conducted on Asian IBD pediatric patients in British Colombia in Canada,^72^ a pattern similar to that of Iranian children vis-à-vis the M/F ratio (male predominance) was observed in both UC and CD patients. In addition, extensive colitis constituted the most frequent form of involvement in the patients. Table 4 depicts a comparison of the epidemiological data between Asian and Iranian IBD patients.

**Table 4:** Comparison of epidemiological aspects of inflammatory bowel diseases between Asia and Iran

**Epidemiological Aspects**	**Asia**	**Iran**
Male/Female ratio	M>F in CD M≈F	M>F in CD F>M in UC
Incidence of EIMs	Low	High
Incidence of PSC in IBD patients	Low	Low
Extent of intestinal involvement (most frequent pattern)	Variable in UC Ileocolic in CD	Proctitis in UC All parts of intestinal lumen in CD
Positive family history	CD>UC	UC>CD
NOD2/CARD15 mutation (in CD)	No relationship	Weak relationship
C3435-T allele (in UC)	Significant relationship	Significant relationship
CTLA-4 gene polymorphism (in UC)	Strong relationship	No Significant relationship

EIMs: Extra-intestinal manifestations; PSC: Primary sclerosing cholangitis; CD: Crohn’s disease; UC: Ulcerative colitis

## Conclusion

The rate of IBDs has increased significantly in Asian countries during the last decade. The most important differences between Asia and Iran in regard to epidemiological aspects are in EIMs, family history, and NOD2/CARD15 mutation in CD patients and CTLA-4 gene polymorphism in UC patients. 

A precise, well-designed, multi-centric, population-based, prospective epidemiologic study must be performed in Asian countries, especially in Iran, in order to shed sufficient light on the incidence and prevalence of IBDs in this region.
